# Germline *MLH1* c.-42 C > T is a likely pathogenic variant predisposing to a reduced-penetrance/modified Lynch syndrome phenotype featuring *MLH1*-methylated cancers

**DOI:** 10.1007/s10689-025-00519-y

**Published:** 2026-01-31

**Authors:** Daniel D. Buchanan, Rocio Alvarez, Khalid Mahmood, Mark Clendenning, Peter Georgeson, Romy Walker, Julia Como, Susan G. Preston, Sharelle Joseland, Kimia Mohammadsaeedi, Francesca Aguirre, Lisa Zhou, Dennis J. Hazelett, Mark A. Jenkins, Christophe Rosty, Ingrid M. Winship, Finlay A. Macrae, Tanya M. Dwarte, Dawn Nixon, Megan P. Hitchins, Jihoon E. Joo

**Affiliations:** 1 Colorectal Oncogenomics Group, Department of Clinical Pathology, The University of Melbourne, Parkville, VIC 3010 Australia; 2 Collaborative Centre for Genomic Cancer Medicine, Victorian Comprehensive Cancer Centre, Parkville, VIC 3010 Australia; 3 Genomic Medicine and Family Cancer Clinic, Royal Melbourne Hospital, Parkville, Melbourne, VIC 3000 Australia; 4 Department of Biomedical Sciences, Cedars-Sinai Medical Center, Los Angeles, CA USA; 5 Melbourne Bioinformatics, The University of Melbourne, Melbourne, VIC 3053 Australia; 6 Colorectal Medicine and Genetics, The Royal Melbourne Hospital, Parkville, VIC 3000 Australia; 7 Department of Medicine, The University of Melbourne, Parkville, VIC 3000 Australia; 8 Department of Computational Biomedicine, Cedars-Sinai Medical Center, Los Angeles, CA USA; 9 Centre for Epidemiology and Biostatistics, Melbourne School of Population and Global Health, Melbourne, VIC 3053 Australia; 10 Envoi Specialist Pathologists, Brisbane, QLD 4059 Australia; 11 University of Queensland, Brisbane, QLD 4072 Australia; 12 Hereditary Cancer Centre, Prince of Wales Hospital, Randwick, NSW Australia; 13 Cancer Genetics Risk Assessment Program, Ascension St. Vincent Health, Indianapolis, IN USA; 14 Department of Cancer Epidemiology, Moffitt Cancer Center, Tampa, FL USA

**Keywords:** Lynch syndrome · *MLH1* methylation · *MLH1* epimutation · ACMG/AMP variant classification · *MLH1* c.-42 C > T · *MLH1* promoter variant

## Abstract

The germline *MLH1* c.-42 C > T (rs41285097) promoter variant has been identified in cases with MLH1-deficient colorectal or endometrial cancers but remains a variant of uncertain significance. Genetic testing identified two new *MLH1* c.-42 C > T index cases from Australia and the USA. Clinicopathologic and molecular characterisation of tumour and non-neoplastic tissues was performed to investigate the potential mechanism of pathogenesis of this variant. The male Australian proband developed MLH1-deficient, *BRAF* p.V600 wildtype, CIMP-negative colon cancer at 61 years. *MLH1* monoallelic methylation and a somatic pathogenic mutation, *MLH1* c.1122_1126dup p.Asp376Valfs*27, were identified in his tumour. Droplet digital PCR (ddPCR) detected mosaic *MLH1* methylation in normal colonic mucosa adjacent to the cancer (3.7%) with lower levels in blood (0.07%) and saliva (0.09%). The USA proband developed MLH1-deficient endometrial cancer at 38 years with *MLH1* monoallelic methylation and loss-of-heterozygosity of the wildtype c.-42 C allele. No evidence of *MLH1* methylation was found by ddPCR in normal tissues. *MLH1* c.-42 C > T heterozygous relatives from both families had either no or extremely low levels of *MLH1* methylation within blood or saliva. Allelic expression from *MLH1* c.-42T was reduced to 70% relative to the wild-type allele in saliva from three heterozygotes. Three additional pedigrees were identified from the Colon Cancer Family Registry. Evaluation of combined multifactorial data from pooled informative index cases supports reclassification of this variant as “likely pathogenic” according to current ACMG/AMP mismatch repair gene-specific guidelines, though with likely reduced penetrance and/or modified phenotype. These findings highlight the clinical importance of identifying *MLH1* c.-42 C > T to inform cancer risk management.

**Supplementary Information:**

The online version contains supplementary material available at 10.1007/s10689-025-00519-y.

## Introduction

Lynch syndrome is caused by a germline pathogenic variant (PV) affecting one of the DNA mismatch repair (MMR) genes (*MLH1*, *MSH2* or *EPCAM*, *MSH6*, and *PMS2*), or more rarely by constitutional *MLH1* methylation (“epimutation”), which confer elevated life-long risks for colorectal, endometrial, and other cancers displaying MMR-deficiency [1]. Non-coding germline variants can cause constitutional *MLH1* methylation, referred to as “secondary *MLH1* epimutation” [2]. Several germline PVs have been associated with secondary *MLH1* epimutations including *MLH1* c.-27 C > A [3, 4], partial promoter deletions/insertions [5, 6], and other complex structural variants [6, 7], which each cause monoallelic methylation of the *MLH1* gene promoter. The c.-11 C > T [8] and c.27G > A [7, 9] variants have been linked to highly variable levels of mosaic constitutional *MLH1* methylation. These contrast with “primary *MLH1* epimutations” where no germline PV appears to be responsible for the constitutional *MLH1* methylation. In carriers of either type of epimutation, subsequent inactivation of the wildtype/unmethylated *MLH1* allele through a somatic second hit in *MLH1*, results in the development of cancers with dual loss of MLH1 and PMS2 protein expression (MLH1-deficiency) [2, 8]. Unlike somatic *MLH1* methylation which commonly underlies sporadic MLH1-deficient colorectal (CRC) and endometrial cancer (EC), constitutional *MLH1* methylation is detectable in tumour and all other normal somatic cells. Because of this, diagnosing carriers of constitutional *MLH1* epimutation involves testing for methylation in non-neoplastic samples (e.g. blood, saliva). *MLH1* epimutation-related cancers, like other Lynch syndrome cancers, present with distinct clinical and molecular features as compared with sporadic *MLH1* methylated cancers, including a younger age at diagnosis [10, 11], and in CRC, absence of both the somatic *BRAF* c.1799T > G (p.V600E) mutation [8, 12] and the disseminated CpG Island Methylator Phenotype (CIMP) [13]. *MLH1* epimutation is estimated to be responsible for up to 75% of CRC cases ≤ 55 years and about 17% of EC cases < 50 years that are MLH1-deficient with *MLH1* methylation in the tumour [11, 14].

A significant proportion of patients with clinicopathologic features of Lynch syndrome do not have an apparent PV or epimutation. A number of these cases carry a variant of uncertain clinical significance (VUS), which complicates genetic counselling and clinical management. Several *MLH1* promoter variants have been identified. Of these, few studies have linked the variant to secondary *MLH1* epimutation as described above, but most remain VUS. The *MLH1* promoter variant c.-42 C > T (Genbank accession NM_000249.4; rs41285097) has been reported in several index cases with suspected Lynch syndrome in the literature [6, 9, 15–17], with multiple additional entries in ClinVar (variation ID: 89593). Currently, the variant is classified as a VUS in ClinVar and has conflicting classifications of pathogenic and VUS in the International Society for Gastrointestinal Hereditary Tumours (InSiGHT) *MLH1* Variant database (both most recently accessed June 19, 2025). Two studies demonstrated that *MLH1* c.-42 C > T resulted in a significant reduction in the transcriptional activity in luciferase promoter reporter assays [9, 16]. One study tested for *MLH1* methylation in peripheral blood leukocyte (PBL) DNA of the proband by methylation-specific PCR, but found none [9]. Another study tested for *MLH1* methylation in the CRC tumour, normal colon tissue, and PBL from the proband using MS-MLPA and MSP, which were all unmethylated [6]. This study also sequenced the cDNA from PBL and found biallelic expression [6]. To determine the potential pathogenicity of *MLH1* c.-42 C > T, we conducted detailed molecular analyses of the tumours and non-neoplastic tissues in two new cancer-affected index cases from Australia and the USA who were heterozygous for this variant. The pedigrees of three additional families harbouring *MLH1* c.-42 C > T from the Colon Cancer Family Registry are also presented. The collated clinicopathologic and disease co-segregation data from these new and previously published cases was used to determine pathogenicity according to revised MMR gene-specific variant classification guidelines by the American College of Medical Genetics and Genomics and the Association for Molecular Pathology (ACMG/AMP).

## Subjects and methods

### Participants and specimens

Two cancer-affected probands underwent germline genetic testing for Lynch syndrome following a diagnosis of MLH1-deficient cancer. The first case (AUS III-2) is a participant of the ANGELS study [18], who was referred from a family cancer clinic in Australia. The CRC-affected mother (AUS II-2) was also heterozygous for the *MLH1* c.-42 C > T variant. The second case (USA Proband) was referred by the genetic counsellor for research-based testing for constitutional *MLH1* methylation from a family cancer clinic in the USA after receiving a negative germline genetic test result by multigene panel testing. First-degree relatives were invited to join the study by the proband or the referring clinical management team. Nucleic acids from blood samples were extracted with the Quick DNA/RNA Microprep plus kit (Zymo Research, Irvine, CA). Saliva and buccal swab samples were self-collected remotely by the USA family using the respective DNA/RNA Shield Collection kits (Zymo Research). Nucleic acids from formalin-fixed paraffin embedded (FFPE) tumour and FFPE adjacent normal tissue were obtained using the AllPrep DNA/RNA FFPE kit (Qiagen, Hilden, Germany). cDNA was synthesized using qScript Supermix (QuantaBio, Beverly, MA).

### Histopathological and molecular testing of CRC and EC tumours

Immunohistochemistry (IHC) of the MMR proteins was performed by the corresponding clinical testing laboratories prior to the recruitment of participants. Where possible, this was confirmed by repeating IHC in our laboratories, as previously described [8, 14]. Genomic DNA (~ 250 ng) was bisulfite converted using the EZ DNA Methylation Lightning Kit (Zymo Research). *MLH1* methylation testing of the promoter C region was performed using Methylight or quantitative CpG pyrosequencing, as previously described [11, 14]. For CRC tumours, CIMP was tested using the five-locus panel (*CACNA1G*, *IGF2*, *NEUROG1*, *RUNX3*, *SOCS1*) by MethyLight [8, 19]. Tumour DNA underwent targeted multigene panel sequencing to assess somatic mutations [18].

### Droplet digital PCR (ddPCR) for the detection of *MLH1* methylation

One of two highly sensitive ddPCR methods was used to assess methylation in the *MLH1* promoter C region in tumour and non-neoplastic tissue specimens. Method one was a methylation-sensitive ddPCR performed on samples from both the Australian family and the USA proband as previously described [8]. The methylation levels were calculated by % $~methylation = number~of$ $\frac{{MLH1~methylated~droplets}}{{MLH1~methylated~droplets + MLH1~unmethylated~droplets}}$ × 100. Method two was a FAM-labelled *MLH1* methylation-specific ddPCR assay duplexed with a HEX-labelled *ACTB* assay, which was used in the USA family only (Supplementary Table 1). Methylation levels were calculated by $\% ~methylation$= $number~of~\frac{{MLH1 - methylated~droplets}}{{ACTB~droplets}}$ × 100%. Both assays have a limit of detection of DNA methylation as low as 0.05% methylation.

### Haplotyping and Ancestry testing across *MLH1*

Genotyping of the promoter c.-93G > A (rs1800734) SNP and c.-42 C > T variants in saliva DNA was performed for each member of the USA family by PCR and Sanger sequencing [3]. Genotyping of the c.655 A > G (rs179997) SNP in the USA family was performed by pyrosequencing, as previously described [20]. To assess shared ancestry between the Australian and the USA cases, six microsatellite regions spanning the *MLH1* locus and beyond (Hg38 chr3:36417631–37377044) were examined using ABI genotyping.

## Functional assessment

### Allelic expression analyses

As previously described [3, 20, 21] and used to measure *MLH1* allelic expression losses and imbalances, heterozygosity at the *MLH1* c.655 A > G SNP within exon 8 was used to determine comparative levels of allelic expression using allele quantification pyrosequencing measured in genomic DNA and cDNA generated from saliva mRNA (USA family) or saliva and PBLs mRNA (healthy subjects), with the allelic expression ratio measured as c.655 A: G in cDNA normalised to genomic DNA.

## Prediction of transcription factor binding changes

The *motifBreakR* software tool was used to interrogate the *Homo sapiens* HOCOMOCO and ENCODE databases to predict alterations in binding motifs for *MLH1* c.-42 C > T, as previously described [22].

### Variant classification

Co-segregation analysis of *MLH1* c.-42 C > T variant (including obligate carriers) and Lynch syndrome-associated cancers was performed using the COOL v3 tool (CO-segregation Online v3, available at http://BJFengLab.org/, accessed March 2025) to obtain the overall Bayes factor score combined from the published and new families in this study. Families were included where phenotype details were provided and the proband plus at least one additional relative (affected or unaffected) had been genotyped for the c.-42 C > T variant. This included obligate heterozygotes. To account for regular colonoscopy screening with polypectomy in relatives, co-segregation analysis was performed using two models where the presence of multiple colorectal polyps; (1) were considered disease-negative, or (2) considered disease-positive.

The cumulative data from cases with the *MLH1* c.-42 C > T variant was used to classify this variant according to the InSiGHT ACMG/AMP MMR gene-specific variant classification criteria version 1.0.0, with the guidelines having recently been approved by ClinGen (available at: https://cspec.genome.network/cspec/ui/svi/doc/GN115, most recently accessed June 5, 2025).

## Results

### Clinicopathologic and molecular findings

*Australian family*

**Fig. 1:**
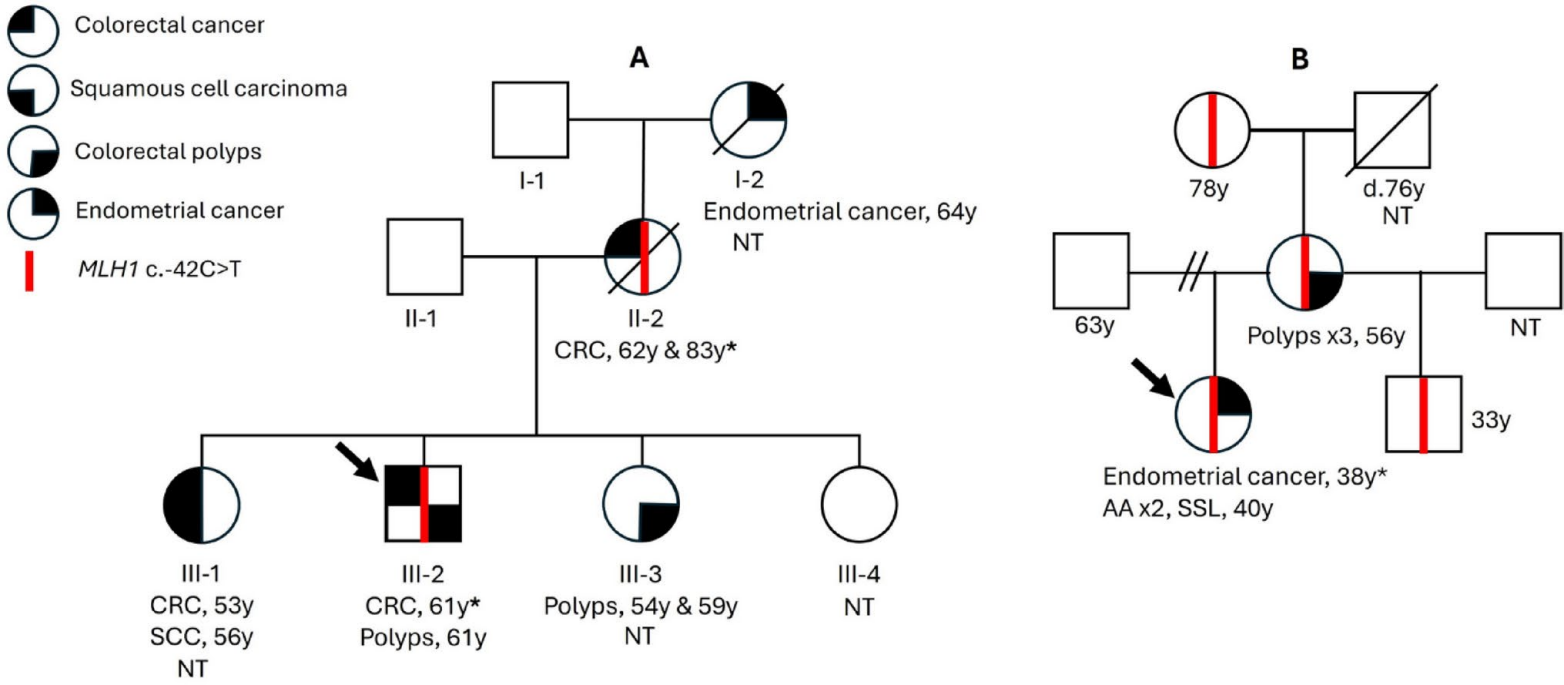
Parsimonious pedigrees of **A** the Australian family and **B** the USA family Arrow: proband. *Samples included in the tumour characterisation. NT, Not tested. CRC, colorectal cancer; SCC, squamous cell carcinoma; EC, endometrial cancer; AA, advanced adenoma; SSL, sessile serrated lesion

The male proband, AUS III-2, developed a low-grade adenocarcinoma of the proximal colon (pT3) at age 61 years (Fig. 1A). The CRC showed loss of MLH1 and PMS2 protein expression by IHC. Tumour *MLH1* methylation testing was positive at 54%. Clinical germline genetic testing identified *MLH1* c.-42 C > T variant as the only candidate causal MMR gene variant. Tumour panel sequencing identified *MLH1* c.1122_1126dup p.Asp376Valfs*27 pathogenic somatic mutation as the likely “second hit”. The tumour was wildtype for *BRAF* c.1799T > A (p.V600E) somatic hotspot mutation and CIMP-negative (Table 1). AUS III-2 had a positive family history of Lynch-spectrum cancers spanning three generations, with four members developing CRC and/or polyps and one with EC (Fig. 1A). In the five affected family members, the mean age at diagnosis of the first CRC/polyps was 58.8 ± 5.0 years (± standard deviation) (Fig. 1A). The proband’s elder sister (AUS III-1) was diagnosed with CRC at 53 years and a younger sister (AUS III-3) was affected with colonic polyps of unknown histology at 54 years, although neither germline genetic nor tumour testing was possible in these sisters. The proband’s mother (AUS II-2) was heterozygous for *MLH1* c.-42 C > T variant and developed metachronous CRCs at 62 and 83 years. The first primary CRC was unavailable for testing. The CRC at 83 years was available and demonstrated loss of MLH1 and PMS2 expression by IHC. The tumour was positive for *MLH1* promoter methylation at ~ 100%. Tumour sequencing identified no somatic mutations or loss-of-heterozygosity (LOH) of *MLH1*. These findings are consistent with biallelic *MLH1* methylation as the cause of MLH1-deficiency. Furthermore, the CRC was CIMP-high and positive for the *BRAF* p.V600E somatic mutation. These findings suggest this CRC developed sporadically via the serrated neoplasia pathway, thus was not causally related to the *MLH1* c.-42 C > T variant.

**Table 1:** The molecular characteristics of the tumours from *MLH1* c.-42 C > T heterozygotes and findings from constitutional *MLH1* methylation testing from the Australian and USA families tested in this study

	AUS III-2	AUS II-2	USA proband
Tumour type	CRC	CRC	EC and metastasis to ovary
Age at Dx years	61	61 ^2^ & 83	38
MMR IHC	MLH1/PMS2 loss	MLH1/PMS2 loss (83yrs); NT (61yrs)	MLH1/PMS2 loss
Tumour *MLH1* methylation ^1^	54%	100%	64%
CIMP status	Negative	High	NA
BRAF p.V600E	Negative (wildtype)	Positive (mutant)	NA
*MLH1* somatic mutation	c.1122_1126dup, p. D376Vfs*27	None detected	LOH of the wildtype c.-42 C allele
ddPCR *MLH1* methylation-sensitive			
Blood	< 1%	NT	0%
Saliva	< 1%	NT	0%
CRC	43.2%	86.2%	NA
Normal colonic mucosa (adjacent to tumour)	3.7%	< 1%	NA
Normal colonic mucosa (resection margin)	0%	0%	NA
Metastatic ovarian tissue	NA	NA	NT
Normal ovarian tissue	NA	NA	0%
ddPCR *MLH1* methylation-specific			
Metastatic ovarian tissue	NA	NA	21%
Normal ovarian tissue	NA	NA	0%

1 MLH1 promoter methylation level as estimated using Methylight technique

2 Tumour tissue was not available for testing

NA=test not applicable; NT=not tested

*USA family*

The USA proband developed a FIGO stage IVB, grade 1 endometrioid adenocarcinoma at 38 years of age presenting with intra-abdominal metastases involving both ovaries and the omentum. No family history of cancer was identified (Fig. 1B). The EC showed loss of MLH1 and PMS2 expression by IHC and was positive for *MLH1* methylation (Table 1). The germline 47 cancer gene panel test (Invitae) had reported no PVs although this test does not interrogate the *MLH1* promoter region for variants other than c.-27 C > A. Sanger sequencing of the *MLH1* promoter in the proband, her mother, half-brother, and maternal grandmother identified the heterozygous *MLH1* c.-42 C > T variant (Fig. 1B). The proband and her mother underwent surveillance colonoscopies, during which two advanced adenomas and a sessile serrated polyp were identified in the proband at age 40 years and three polyps of mixed histology were identified in the mother at age 56 years.

**Fig. 2:**
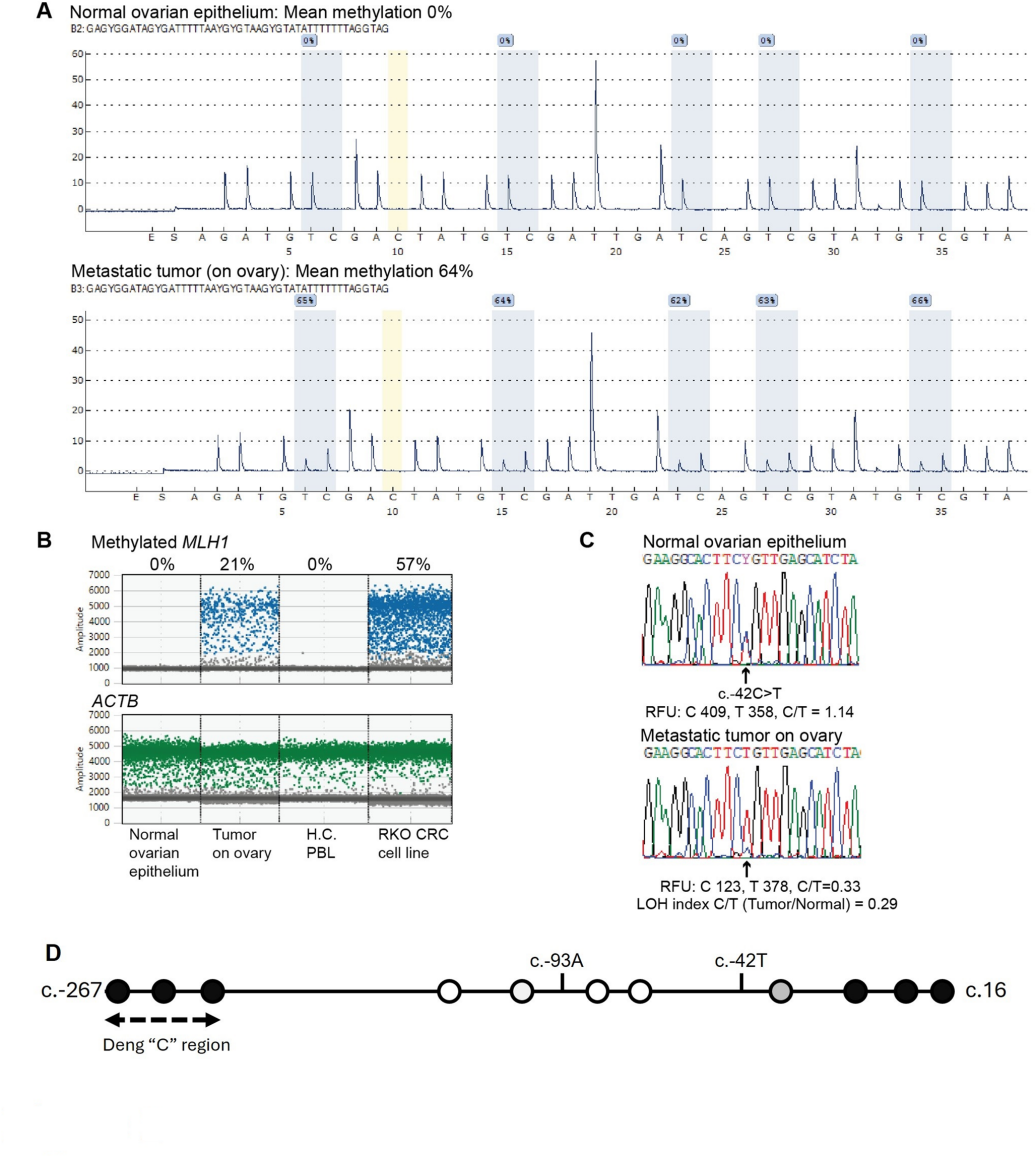
*MLH1* promoter methylation accompanied by loss-of-heterozygosity of the wild-type allele at *MLH1* c.-42 C > T in the metastatic endometrial cancer of the USA proband. Quantitation of *MLH1* promoter methylation in formalin-fixed paraffin-embedded (FFPE) metastatic tumour (ovarian lesion) and uninvolved normal ovarian tissue samples by** A** CpG pyrosequencing and** B**, droplet digital methylation-specific PCR, including healthy control (H.C.) leukocyte (PBL) and RKO colorectal cancer (CRC) cell line as methylation negative and positive controls, respectively. **C,** Sanger sequencing across the *MLH1* c.-42 C > T site shows loss-of-heterozygosity (LOH) of the c.-42 C allele in the tumour. RFU, relative fluorescence units. **D,** pictogram showing pattern of allelic and individual CpG methylation detected in tumour DNA by Sanger sequencing of methylation-specific PCR amplicons. Circles represent CpG sites, with unmethylated shown in white, hypermethylated in black, and partially methylated in gray. Methylation was confined to the c.-[93 A; 42 T] haplotype, although not in the immediate vicinity of c.-42 C > T. Dotted line indicate the Deng “C” region [23] assessed by Methylight and methylation-sensitive ddPCR

In the metastatic lesion on the ovary, *MLH1* methylation was detected at 64% by pyrosequencing (Fig. 2A), and 21% by *MLH1* methylation-specific ddPCR (Fig. 2B) with LOH of the wildtype c. -42 C allele identified (Fig. 2C). Although the allelic methylation status could not be assessed directly at the c.-42 C > T on the sense strand (since the bisulfite conversion process renders unmethylated cytosines indistinguishable from thymines), we were able to assess the methylation status indirectly using the nearby c.-93 G > A promoter SNP, for which the proband was also heterozygous (Supplementary Table 2). Segregation analyses in the nuclear family had shown the c. -93 A allele was linked to the c.-42 T genotype generating a variant c. -[93 A;42 T] haplotype. The methylation-specific PCR assay that flanks both c.-93 G > A and c.-42 C > T followed by Sanger sequencing [14], identified methylation on the c.-42 T variant haplotype, which was retained in the tumour (Fig. 2D; Supplementary Fig. 1). The methylation pattern across the variant haplotype showed that the CpGs immediately flanking c. -42 T were unmethylated or only partially methylated, while the CpGs further upstream and downstream were fully methylated (Fig. 2D; Supplementary Fig. 1). The Deng C regulatory region [23] situated upstream of the c.-42 site was methylated in the tumour sample.

### Assessment of *MLH1* methylation in non-neoplastic samples

**Fig. 3:**
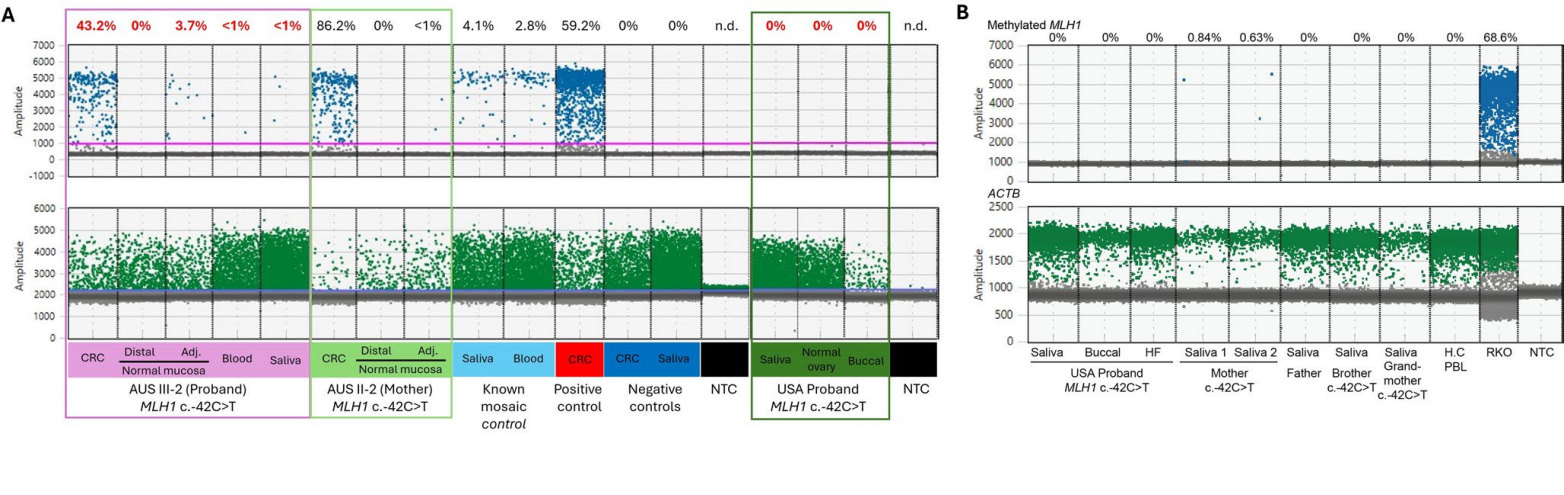
Amplitude plot of *MLH1* methylation-sensitive ddPCR in CRC and normal tissue samples from the AUS participants and the USA proband Blue dots indicate “droplets” containing positive methylation (FAM) whereas green dots indicate droplets containing unmethylated DNA (HEX). **A** Column 1–5 are samples from AUS III-2; column 6–8 from AUS II-2 and column 15–17 are from USA Patient. DNA from saliva and blood from a known mosaic *MLH1* methylation case (Joo et al. 2023), DNA from sporadic *MLH1* methylated CRC (positive control), sporadic *MLH1* unmethylated CRC and saliva from unrelated individual (negative control) was included as controls. Distal normal colonic mucosa denotes normal mucosa tissue collected from the resection margin of the tumour and adj. (adjacent) normal mucosa was collected from normal tissue samples adjacent to the tumour. NTC - no template control. **B** Amplitude plot of *MLH1* methylation-specific ddPCR duplexed with *ACTB* in normal tissue samples from the USA proband and her first-degree relatives. Blue dots indicate “droplets” containing positive *MLH1* methylation (FAM) whereas green dots indicate droplets containing *ACTB* (HEX) as input control. H.C. PBL, healthy control leukocyte DNA (negative control), RKO, methylated colorectal cancer cell line DNA (positive control). The mother was the only *MLH1* c.-42 C > T heterozygote in whom extremely low level (< 1%) *MLH1* methylation was detected in saliva DNA

Given the findings of tumour *MLH1* methylation, we tested for constitutional *MLH1* methylation in non-neoplastic samples from c.-42 C > T heterozygotes using methylation-sensitive ddPCR. Proband AUS III-2 had low-level methylation in normal colonic mucosa adjacent to the tumour (3.7%), and extremely low levels in blood (< 1%) and saliva (< 1%), but no methylation (0%) was detected in normal colonic mucosa collected from the resection margin distant from the tumour (Table 1; Fig. 3A). In AUS II-2, only the CRC, and the normal mucosa from the resection margin and adjacent to the tumour tissue were available for testing as the participant was deceased. DNA from the normal colonic mucosa adjacent to the tumour showed < 1% methylation, but DNA from normal mucosa at the distant resection margin was unmethylated (0%) (Table 1; Fig. 3A). The USA patient showed no evidence of *MLH1* methylation in saliva, normal ovarian epithelium (adjacent to the metastatic ovarian tumour), or buccal DNA samples (Table 1; Fig. 3A).

Additional *MLH1* methylation-specific ddPCR testing combined with *ACTB* was performed in the saliva, normal ovarian epithelium and buccal DNA samples from the USA proband, which were also unmethylated (Table 1; Fig. 3B). Extremely low-level (< 1%) of *MLH1* methylation was detected in the saliva DNA from the mother, which was confirmed in a second DNA extraction of the same saliva sample (Fig. 3B). *MLH1* methylation was not identified in the saliva DNA samples from the other two *MLH1* c.-42 C > T heterozygous relatives from the USA family (Fig. 3B).

### Shared ancestral haplotype between the two new index cases

To examine shared ancestry between the Australian and USA probands, we assessed length variation in six highly polymorphic microsatellite regions within or flanking the *MLH1* locus. The AUS III-2 and USA probands had identical lengths in five contiguous polymorphic regions spanning at least 462 kb (chr 3: 36955843–67418535), suggesting a high likelihood of shared ancestry between these two index cases (Supplementary Table 2). Genotyping and segregation analyses of the *MLH1* c.-93G > A and c.655 A > G SNPs in the USA family showed the haplotype linked to the variant c.-42 C > T allele was c.[-93 A;-42 T;655 A] (Supplementary Table 2).

## Assessment of the functional impact of *MLH1* c.-42 C > T

### Comparison of allelic expression levels

**Fig. 4:**
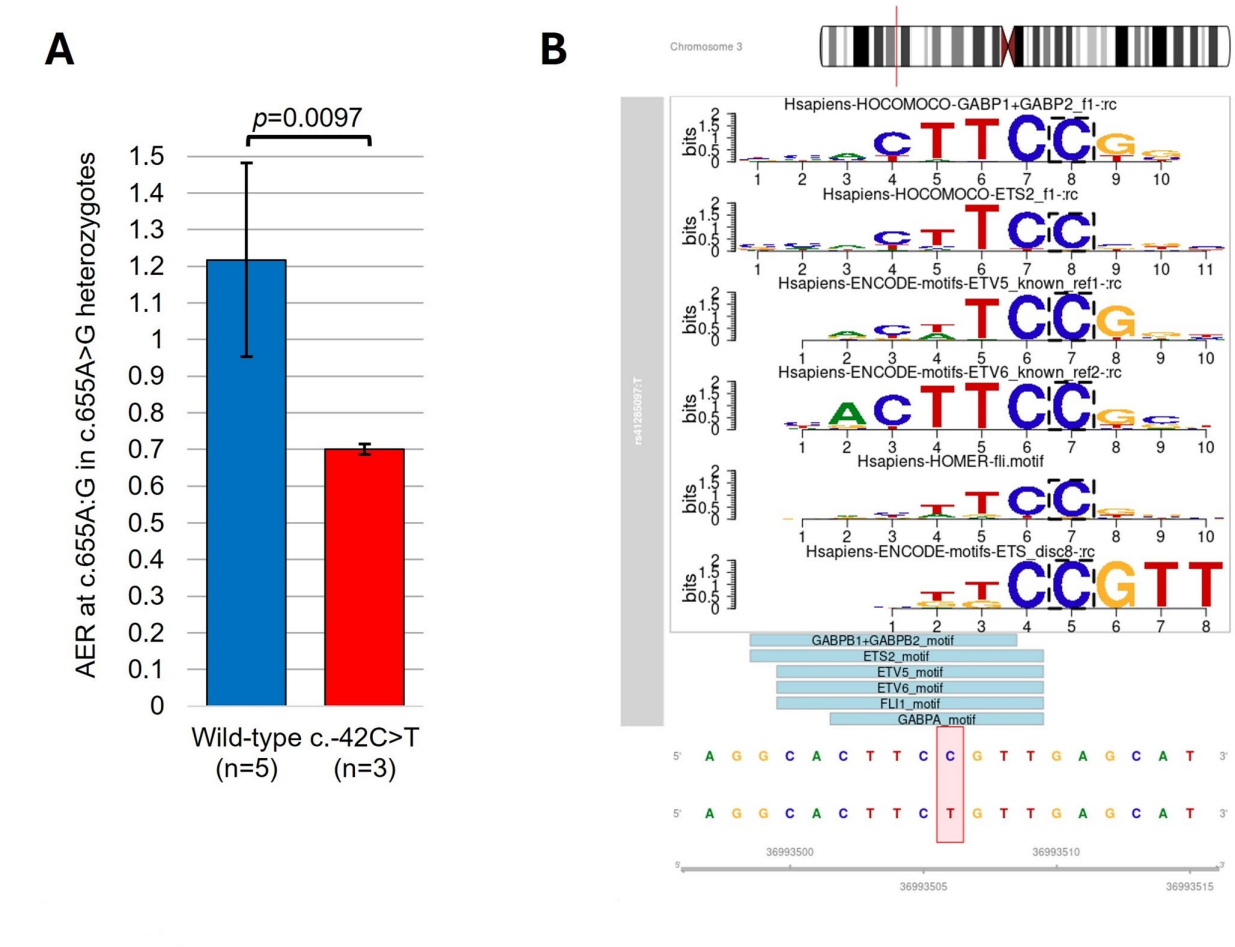
Functional impact of *MLH1* c.-42 C > T. **A.** Allelic expression from the MLH1 c.-42C>T variant haplotype was reduced on average to 70% of the wildtype haplotype in cDNAgenerated from saliva mRNA from three USA family members (mother, grandmother, and half-brother) who were dual heterozygotes for the MLH1 c.-42C>T variant and exon 8 c.655A>G SNPat which allelic expression ratios (AERs) were measured by allele quantitative (AQ)pyrosequencing. The variant MLH1 haplotype was c.[-42T;655A], therefore, AERs weremeasured as c.655A:G. This difference was statistically significant as compared to five healthysubjects (four blood leukocytes, one saliva) who were wild-type MLH1 c.-42C and heterozygousc.655A>G (student’s two-tailed t-test assuming equal variance). See Supplementary Figure 2 for an example of AQ pyrosequencing in the USA family.** B.** Altered transcription factor (TF) binding predicted by MotifBreakR. Consensus motifs for TFs (as labelled) broken by the C>T transition are shown in colour above. Below, bars show the sequence across which the respective TFs arepredicted to bind, based on ChIP-seq data in the HOCOMOCO and ENCODE databases. See accompanying Supplementary Table 3 for statistics

The USA proband was homozygous A at the expressed c.655 A > G SNP in *MLH1* exon 8, therefore, was uninformative for allelic expression analyses (Supplementary Table 2). However, three family members (mother, maternal grandmother, and half-brother) were dual *MLH1* c.-42 C > T and c.655 A > G heterozygotes, with known haplotypes c.[-42 C; 655 G] and c.[-42 T; 655 A], thus were assessable for allelic expression imbalance using a pyrosequencing assay previously applied for this purpose [3, 21]. Allelic expression ratios measured in saliva from the three relatives showed the c.655 A allele linked to c.-42T was expressed at an average of 70% relative to the c.655G allele linked to c.-42 C (Fig. 4A and Supplementary Fig. 2). This reduced level of allelic expression was statistically significant when compared to five wild-type c.-42 C healthy subjects heterozygous at c.655 A > G, among whom the c.655 A allele was expressed at an average of 122% of the c.655 G allele in PBLs (*n* = 4) and saliva (*n* = 1) (Fig. 4A).

### Identification of changes in transcription factor binding

*MotifBreakR* analysis was used to identify changes in transcription factor binding based on prior ChIP-seq data in the HOCOMOCO and ENCODE databases. This showed the *MLH1* c.-42 C position represents a binding site for six major transcription factors and the C > T substitution is predicted to reduce their binding (Fig. 4B and Supplementary Table 3).

#### Additional index cases with *MLH1* c.-42 C > T from the Colon Cancer Family Registry

Three new index cases heterozygous for the *MLH1* c.-42 C > T variant were identified from the Colon Cancer Family Registry (Table 2). One was a male proband with an unremarkable family history who developed CRC at 44 years of age (Supplementary Fig. 3). The second proband was a female who developed CRC at 69 years and had a positive family history of cancer, including CRC in relatives presenting in their sixties. The CRC of this proband and her sister were both *MLH1* methylated (Supplementary Fig. 4). The third was a male proband diagnosed with sigmoid colon cancer at 46 years with multiple cancer affected siblings (Supplementary Fig. 5). For all three index cases, no tissue samples were available for further molecular testing.

**Table 2:** Summary of *MLH1 *c.-42 C > T heterozygotes from previous reports and the current study where phenotypic data were provided

Study	Proband/Relationship	Person ID	Country	Race/ ethnicity	Sex	Neoplasms (Dx Age)	MMR IHC	Tumour MLH1 methylation	Clinical criteria met		Included in segregation analysis
Green et al. [16]	Propositus	“IV:1”	Canada	European	F	Not reported	NA	NA		Amsterdam I	Yes
	Father	“III:2”	Canada	European	M	Colonic polyps (66y & 68y)	Not tested	Not tested			Yes
	Uncle (paternal)	“III:1”	Canada	European	M	CRC (76y)	MLH1 loss	Not tested			Yes
	Cousin (paternal)	“III:7”	Canada	European	M	CRC (35y)	Not tested	Not tested			Yes
Mangold et al. [15]	Index	NA	Germany	NA	NA	CRC (unknown)	MSI-H	Not tested		Rev. Bethesda+	No
Ward et al. [9]	Index	“J”	Seattle	European	F	CRC (53y)	MSI-H MLH1-loss	Not tested		Rev. Bethesda	No
Morak et al. (6)	Index	NA	Germany	European		CRC (unknown)	MSI-H MLH1-loss	Negative		Unknown	No
Buchanan et al.	Index	“AUS III-2”	Australia	European	M	CRC (61y)	MLH1/PMS2 loss	Positive		Rev. Bethesda	Yes
	Mother	“AUS II-2”	Australia	European	F	CRC (61y & 83y)	MLH1/PMS2 loss (colon 83y)	Positive			Yes
	Index	“USA proband”	USA	European	F	Endometrial cancer (38y)	MLH1/PMS2 loss	Positive		Rev. Bethesda	Yes
	Mother		USA	European	F	Colonic polyps (56y)	Not tested	Not tested			Yes
	Index (CCFR)	NA	Australia	European	M	CRC (44y)	Not tested	Not tested		Rev. Bethesda	Yes
	Index (CCFR)	NA	Canada	Native American	M	CRC (47y)	Not tested	Not tested		Rev. Bethesda	No
	Index (CCFR)	NA	USA	European	F	CRC (69y)	MLH1/PMS2 loss	Positive		Rev. Bethesda	Yes
	Sister	NA	USA	European	F	CRC (64y&81y)	MLH1/PMS2 loss	Positive			Yes
	Brother	NA	USA	European	M	NA	NA	NA			Yes

Dx Age = Age at diagnosis in years; MMR IHC = DNA mismatch repair immunohistochemistry; CCFR- Colon Cancer Family Registry; NA=not available; F=female; M=male; CRC = colorectal cancer; Rev.= Revised Bethesda; +=Bethesda or more stringent criteria used for patient inclusion in study

## Multifactorial evidence for classification of *MLH1* c.-42 C > T

To classify the *MLH1* c.-42 C > T variant, we combined informative data from the previously reported and new index cases reported herein (Table 2). We evaluated the clinical and molecular phenotypes, impact on gene function, population frequencies, and co-segregation with disease in (eligible) familial cases in accordance with the InSiGHT ACMG/AMP MMR gene-specific variant classification guidelines. The assessment of phenotypic, family history, and genetic data supported the evidence of pathogenicity with a score of “PP4_Strong” on account of multiple independent tumours demonstrating MLH1-deficiency among several families, with *MLH1* c.-42 C > T being the sole candidate causal MMR gene variant identified in each case (Table 3). The co-segregation analysis revealed a score of “PP1_Moderate” when considering Lynch syndrome-associated cancers only or “PP1_Strong” when incorporating multiple colonic polyps as disease-positive in the analysis (Table 3). The assessment of population frequency scored “BS1_Strong”, based on the Grpmax Filtering Allele Frequency of 0.0001291 (within the ≥ 0.0001 to < 0.001 range) in the gnomAD v4.1 dataset (most recently accessed June 9, 2025) [24]. This score is based on the assumption that *MLH1* c.-42 C > T is not a founder PV, which we cannot rule out. Collectively, combining the “PP4_Strong” and “PP1_Moderate” scores supports reclassification of *MLH1* c.-42 C > T variant as “likely pathogenic” when using the results from co-segregation analysis incorporating cancer diagnoses only. Alternatively, combining “PP4_Strong” and “PP1_Strong” scores supports reclassification of the *MLH1* c.-42 C > T variant as “pathogenic” when incorporating multiple colonic polyps in the disease phenotype.

**Table 3:** Multifactorial evidence for the reclassification of *MLH1* c.-42 C > T. Evidence in key areas of consideration was assessed for reclassification of *MLH1* c.-42 C > T according to the current MMR gene-specific guidelines by the international society for Gastrointestinal hereditary tumours (InSiGHT) American College of Genetics and Genomics and Association for Molecular Pathology (ACMG/AMP) scoring system. The points of evidence for pathogenicity including one “Strong” score in the phenotype category, and either one “Strong” or one “Moderate” score in the disease co-segregation category, meet the criteria for classification as a “likely/pathogenic variant”, in the absence of sufficient functional evidence and notwithstanding a population frequency marginally higher than 0.0001 (0.01%). ^1^

Evidence	ACMG/AMP score
*Phenotype*	
Patient phenotype and family history of Lynch syndrome identified in ≥ 3 independent CRC/Endometrial tumours with MSI and MLH1-loss in ≥ 2 families is specific for the gene in which the variant was identified and no other MMR gene candidate variant was identified.	PP4_Strong
Presence of MLH1 methylation in the tumour was not considered a criterion for exclusion given the hypothesis of low level of constitutional MLH1 methylation.	
*Function*	
Partial reduction in gene expression is insufficient for scoring (i.e. this was > 10% of wildtype cDNA)	Not scored
*Co-segregation with disease*	
COOL v3 analysis incorporating colonic polyps in disease phenotype	PP1_Strong (incorporating polyps)
* Co-segregation overall LOD score: 1.33406*	
* Co-segregation overall Bayes factor: 21.58043*	
COOL v3 analysis cancers only	PP1_Moderate (cancers only)
* Co-segregation overall LOD score: 0.75034*	
* Co-segregation overall Bayes factor: 5.62784*	
*Population frequency*	BS1_Strong, but variant cannot exclude as a founder pathogenic variant
gnomAD v4.1^1^ dataset: Grpmax filtering allele frequency (AF) 0.0001291; highest AF 0.0001476 in (non-Finnish) Europeans, falls within the ≥ 0.0001 and < 0.001 range	
*Combined criteria*	≥ 2 Strong (PP4, PP1) = Pathogenic 1 Strong (PP4) and 1 Moderate(PP1) = LikelyPathogenic
Cancers and colonic polyps	
Cancers only	

## Discussion

Our study provides a detailed molecular investigation of two index cases (AUSIII-2 and USA) heterozygous for the *MLH1* c.-42 C > T promoter variant. To our knowledge, we report the first observations of tumour *MLH1* methylation in heterozygotes of the *MLH1* c.-42 C > T variant, as observed in both AUS III-2 and USA probands, as well as two members of a family from the CCFR. Our tumour findings from the AUS III-2 and USA index cases are reminiscent of cases with mosaic “secondary *MLH1* epimutations”, wherein we observed amplified tumour *MLH1* methylation accompanied by a somatic second hit in *MLH1* inactivating the c.-42 C wildtype allele, and the absence of the *BRAF* p.V600E mutation and CIMP in the CRC from AUS III-2. In the USA proband, we observed monoallelic methylation specifically of the c.-42 T allele, accompanied by LOH of the c.-42 C (wildtype) allele. Tumourigenesis thus appears to have been driven by the acquisition of a somatic “second hit” in *MLH1* in cells-of-origin that contained an epigenetically inactivated copy of the *MLH1* c.-42 T allele as the “first hit”, which led to a MLH1-deficient cancer displaying amplified *MLH1* methylation, even though methylation was undetected or extremely low in normal tissues.

The cumulative evidence from tumour phenotype and co-segregation analysis of the variant from the previously reported cases and those reported in this study support re-classification of this variant as likely pathogenic. The c.-42 C > T variant likely affects transcription factor binding with evidence of reduced expression of the variant allele. The two families we studied in detail displayed some evidence of shared ancestry, with an overlapping haplotype spanning at least 462 kb across *MLH1*. Our study did not exclude the possibility that another causal genetic variant may lie within this shared region of overlap. For example, previous studies have reported structural and copy number variations linked to secondary *MLH1* epimutation that were only detected using long-range sequencing methods [7, 25]. Although our targeted multigene NGS covered the complete *MLH1* gene, including 1 kb upstream of the transcription start site and intronic regions, this technology is not designed to identify complex genetic alterations. Due to sample insufficiency (e.g. the USA proband had only limited saliva or FFPE DNA samples), we were unable to apply long-read sequencing technologies (e.g. Oxford Nanopore, PacBio). Extended sequencing in families bearing *MLH1* c.-42 C > T would be of interest in future to comprehensively investigate the possibility of a linked causal variant as well as the extent of shared ancestry between families [26].

While the molecular findings from the tumour analysis in the two probands collectively suggest focal, monoallelic methylation of the *MLH1* c.-42T allele contributing to tumourigenesis, the timing of the methylation event from non-neoplastic tissue to neoplasia remains in question. Extremely low levels of methylation (< 1%) were inconsistently identified in the PBL and/or saliva DNA of only two heterozygotes. In two heterozygotes in the Australian family, *MLH1* methylation was detected at low (3.7%) or extremely low (< 1%) levels only in normal colonic mucosa adjacent to their respective tumours. Whilst mosaic *MLH1* epimutation localised to the specific location of the colon is possible, this remains inconclusive as potential contamination from the adjacent cancer cannot be obviated. Interestingly, the c.-42 T allele in the metastatic tumour sample of the USA proband showed highly methylated CpG sites either side of a short region in the immediate vicinity of the c.-42 T site that was unmethylated or only partially methylated. This precise gap in methylation is unlikely to have been an artefact of the assay, since the same methylation-specific PCR assay has been used to assess allelic *MLH1* methylation in previous patients with low-level mosaic constitutional *MLH1* methylation including the c.-42 region [14]. One possibility is that a nuclear factor bound to the c.-42 C > T site may have occluded the region and prevented methyltransferases from accessing those CpG sites. Nevertheless, the c.-42 is situated outside the Deng “C” region, where methylation has the highest regulatory impact [23]. Our findings differ from a previously reported case in whom neither constitutional, nor tumour *MLH1* methylation was identified [6], although the tumour was not further characterised to determine if this locus was intact. In another previously reported case, LOH was observed, but this was of the *MLH1* c.-42 T allele [16]. More detailed molecular pathologic characterisation of the tumours from other index cases with *MLH1* c.-42 C > T is warranted to determine its mechanism of pathogenesis.

We demonstrated a consistent reduction in expression of the *MLH1* c.-42 T allele to 70% of the wild-type allele in saliva (non-neoplastic) samples from three heterozygotes in the USA family who were all unaffected by cancer. We were unable to determine the extent of allelic expression reduction in other sources of normal tissues due to sample limitations. Nevertheless, to our knowledge, these are the first observations of reduced allelic expression from the c.-42 C > T variant in any tissue samples from heterozygotes. A prior study found biallelic expression by sequencing cDNAs from both *MLH1* and *EMP2AIP1* (bidirectional genes that share the same CpG island promoter) in PBL from their proband [6]. However, this assay may not have been sufficiently sensitive to detect a subtle allelic expression imbalance. Our findings are consistent with prior promoter reporter assays by us and others, although the relative levels of transcriptional output from the c.-42 T promoter constructs from 37% to 85% relative to the wildtype constructs, indicating wide inter-assay or cell context-dependent variability [9, 16]. This reduced allelic expression may be variably mediated by reduced binding of transcription factors including c-Myb [16], GABPA, FLI1, and ETS, in different tissue contexts, each of which were predicted to have altered interactions. This site is overrepresented with “transcriptionally active” H3K27 acetylation marks [27] and CAGE reads [28], further supporting its regulatory importance. The precise mechanism by which *MLH1* c.-42 C > T results in reduced transcription remains to be confirmed in functional assays, which are beyond the scope of this study.

A key phenotypic feature of *MLH1*-Lynch syndrome and previously identified *MLH1* epimutation cases has been a young age at CRC or EC diagnosis [10]. The age at cancer diagnosis was seemingly older for the *MLH1* c.-42 C > T variant carriers with only 4/10 cancer-affected carriers diagnosed < 50 years of age (Table 2) [9, 16]. Whilst the ages at first diagnosis of CRC are later than the average age of diagnosis typically reported in Lynch syndrome, they are also significantly earlier than sporadic *MLH1* methylated and CIMP-high CRCs [29, 30]. This intermediate age at cancer diagnosis may be explained by the partial reduction in expression from the variant allele, rather than a complete loss of protein function in cases with a classic PV or complete allelic silencing seen in *bona fide* epimutation cases [31].

We collated multifactorial data from the new families reported herein combined with previously reported cases to determine if there was sufficient evidence to support a definitive reclassification of the *MLH1* c.-42 C > T variant in accordance with ACMG/AMP MMR gene-specific guidelines released in 2024. Phenotypic and genetic evidence in favour of pathogenicity were strong, given *MLH1* c.-42 C > T was the sole candidate variant identified in clinical genetic testing likely to underlie the MLH1-deficient phenotype that has now been observed in multiple independent tumours in several families. However, these guidelines state that *MLH1* promoter methylation is to be excluded in the tumours under the presumption that all *MLH1*-methylated tumours are sporadic in occurrence (as opposed to Lynch syndrome-related). While this is true in most cases, we suggest an exception clause when considering cases with *MLH1* promoter or other variants that may underlie “secondary *MLH1* epimutations”. Co-segregation data provided “Moderate” or “Strong” evidence in favour of a pathogenic role for c.-42 C > T in the development of cancers only, or cancers plus multiple polyps, respectively. Thus, according to the rules for combining criteria, between the phenotypic and co-segregation categories, the scores met the criteria for reclassification of *MLH1* c.-42 C > T as likely pathogenic (cancers only) or pathogenic (polyps included). Whether pathogenic or likely pathogenic, these classifications are both clinically actionable.

The functional category of the ACMG/AMP MMR gene-specific guidelines for the assessment of pathogenicity could not be scored based on the partial loss of transcription observed in normal tissues nor of allele-specific methylation in the tumour. Therefore, function did not contribute for or against pathogenicity. A further confounding factor in this assessment was the scoring based on population frequency. The allele frequency (AF) in gnomAD v4.1 across all populations was 0.0001291, marginally higher than the threshold AF of 0.0001 (0.01%), therefore, considered as a common variant. This AF fell into the benign scoring range, provided the variant is excluded as a founder PV, which we cannot exclude. Indeed, based on the shared haplotype spanning at least 462 kb across *MLH1* in the two seemingly unrelated families from different continents, there does appear to be shared ancestry, as was the case for the *MLH1* c.-27 C > A variant [4]. However, the full extent of haplotype sharing between these two families was not explored. All cancer-affected *MLH1* c.-42 C > T cases reported to date have been of European heritage and the highest AF of 0.0001476 has been observed in the European (non-Finnish) population. The gnomAD v4.1 datasets report very low AFs in the Admixed American (0.00003332) and African American (0.00001338) populations, which are likely derived from European-origin admixtures since this allele was absent in African and other populations. Collectively, it is possible that *MLH1* c.-42 C > T is indeed a European founder PV with reduced penetrance on account of the partial allelic loss of expression, however, extended genotype analyses in these and other families with this variant are required to provide definitive evidence for this.

The penetrance of the *MLH1* c.-42 C > T variant remains an important outstanding question. We were unable to assess this, given the small number of families studied herein and reported to date. Our study objective was to assess the clinicopathologic and functional evidence for pathogenicity of the variant within the presenting cases and this remains a limitation. Nevertheless, the wide variation in age of cancer onset, the observed partial loss of allelic expression in mRNA, and the relatively high population frequency of the variant, collectively suggest *MLH1* c.-42 C > T is a moderately penetrant variant, as has previously been described for other *MLH1* founder variants [32]. Furthermore, CRC and endometrial cancer were the only cancers within the broader Lynch syndrome spectrum reported in the *MLH1* c.-42 C > T families, suggesting this variant confers a modified *MLH1*-Lynch syndrome phenotype. This may also be a consequence of reduced penetrance, but may also reflect potential differences in allelic expression levels between tissues. Further assessment within individual families and more broadly of in larger, independent cohorts of cancer cases as well as the general population, will be important to define the penetrance and phenotypic spectrum associated with the *MLH1* c.-42 C > T variant.

In conclusion, the cumulative and multifactorial evidence support the classification of the *MLH1* c.-42 C > T variant as likely pathogenic for *MLH1*-Lynch syndrome, notwithstanding its unscorable functional evidence and benign threshold population frequency. In the USA, while part of the *MLH1* promoter region is technically covered by most next-generation sequencing panels, it is not bioinformatically interrogated other than at *MLH1* c.-27 C > A in a site-specific manner [3, 4, 9]. Thus, most variants within the *MLH1* promoter region are currently not reported largely due to their classification as clinically non-actionable VUS. We proffer that *MLH1* c.-42 C > T should also be included and reported in routine genetic testing of patients with MLH1-deficient cancers who are suspected to have Lynch syndrome, including those whose tumour shows *MLH1* methylation.

## Supplementary Information

Below is the link to the electronic supplementary material.

Supplementary Material 1

*MLH1* promoter methylation affects the *MLH1* c.-[93A;42T] haplotype in the metastatic endometrial cancer of the USA proband (also see Figure 2D).Top: Sanger sequencing across a fragment of the *MLH1* promoter region in untreated peripheral blood leukocyte (PBL) genomic DNA of the USA proband shows heterozygosity for the c.-93G>A SNP and the c.-42C>T single nucleotide variant. Based on segregation analyses in her parents, the two haplotypes are wild-type c.-[93G;42C] and variant c.-[93A;42T]. The region shown contains 12 potential CpG sites (underlined), of which one at c.-42 is present only on the wild-type c.-42C allele (*).Beneath: Sanger sequencing of the corresponding promoter region amplified by methylation-specific PCR from bisulfite-converted DNA extracted from the metastatic endometrial tumour. The c.-42C>T site is indistinguishable in bisulfite-converted DNA since the bisulfite conversion process convert unmethylated cytosines to uracils, which are then sequenced as thymines (T), whereas methylated cytosines (C) remain unconverted. Only the c.-[93A;42T] haplotype was amplified, indicating methylation was confined to this haplotype. Therefore, the methylation signal in the tumour was derived from the retained variant c.-42T allele (with LOH of the wild-type c.-42C allele). Interestingly, the CpG sites immediately flanking c.-42T were unmethylated only partially methylated, whereas CpG sites further upstream and downstream were hypermethylated.

Supplementary Material 2

Example of allele quantitation pyrosequencing at *MLH1* exon 8 SNP c.655A>G showing allelic expression imbalance in a dual heterozygote for c.-42C>T and c.655A>G from the USA family.The grey shaded area shows the relative quantification of the A and G alleles at the c.655 site in saliva DNA (top) and saliva mRNA (bottom) from the half-brother of USA proband, with percentage levels of each allele measured in coloured boxes above. AER was calculated for A:G , given the c.655A allele was linked to the c.-42T allele on the same haplotype.

Supplementary Material 3

Supplementary Material 4

Supplementary Material 5

Supplementary Material 6

## Data Availability

Sanger sequencing, pyrosequencing, and next-generation sequencing data will be made available upon special request to the authors.
