# Supplementary figures and images for: Germline MLH1 c.-42 C > T is a likely pathogenic variant predisposing to a reduced-penetrance/modified Lynch syndrome phenotype featuring MLH1-methylated cancers
Source: Fam Cancer. 2026 Jan 31;25(1):17. doi: 10.1007/s10689-025-00519-y (PMC12860845; doi:10.1007/s10689-025-00519-y)

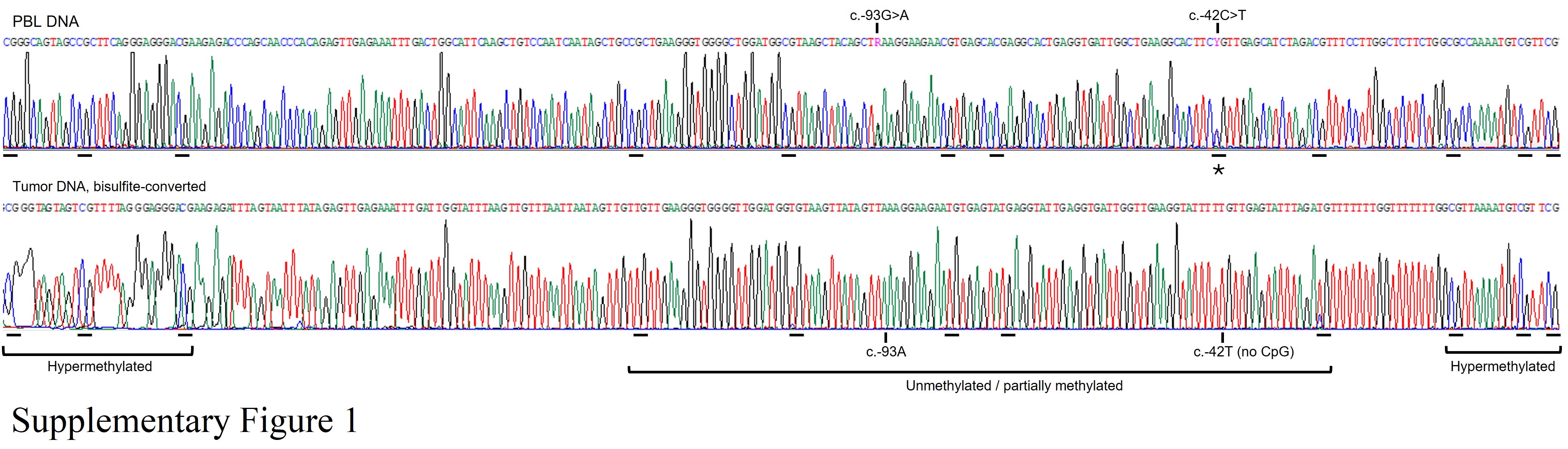

Supplement: Supplementary file 1 — Supplementary Material 1 [file 10689_2025_519_MOESM1_ESM.jpg]

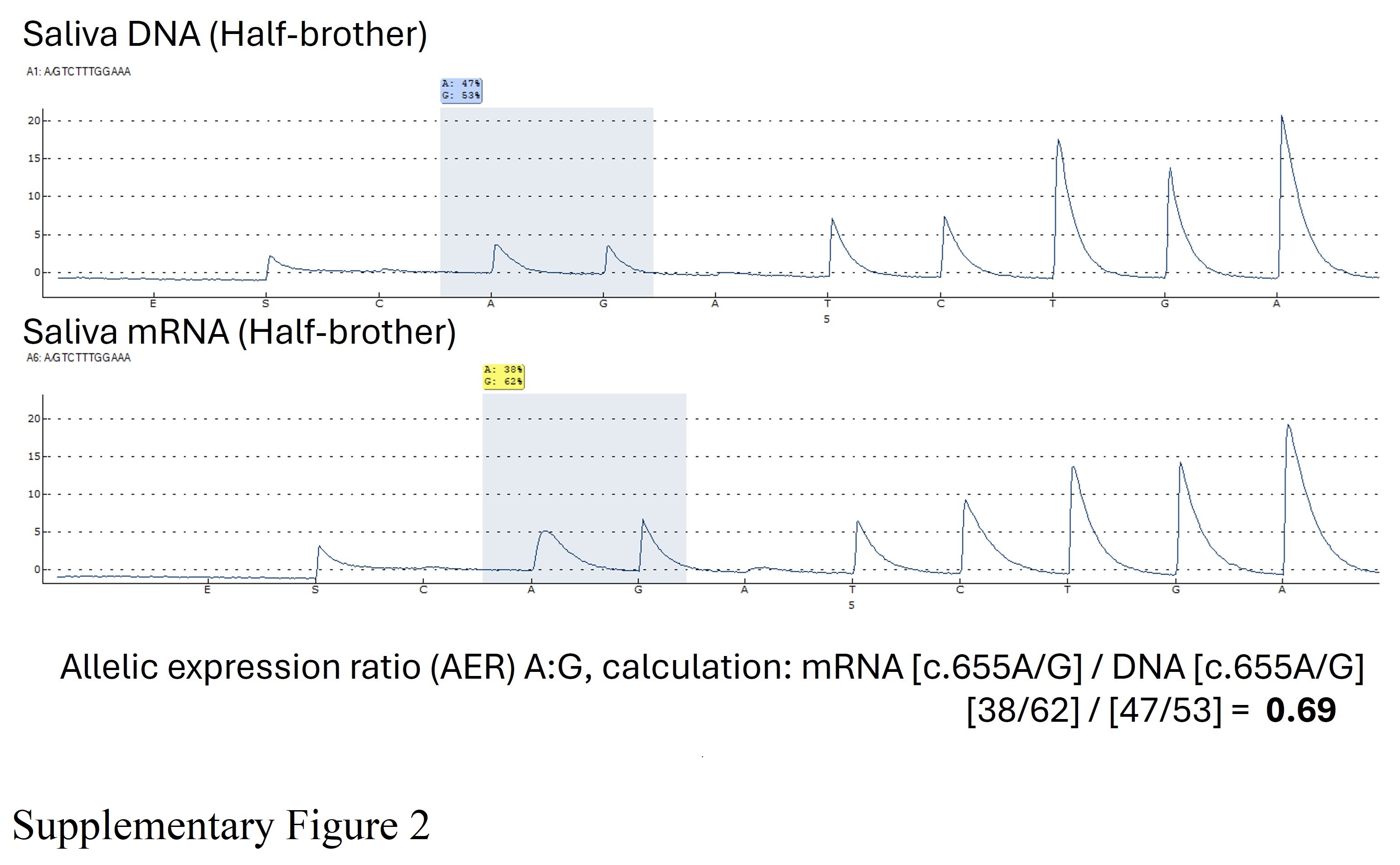

Supplement: Supplementary file 2 — Supplementary Material 2 [file 10689_2025_519_MOESM2_ESM.jpg]

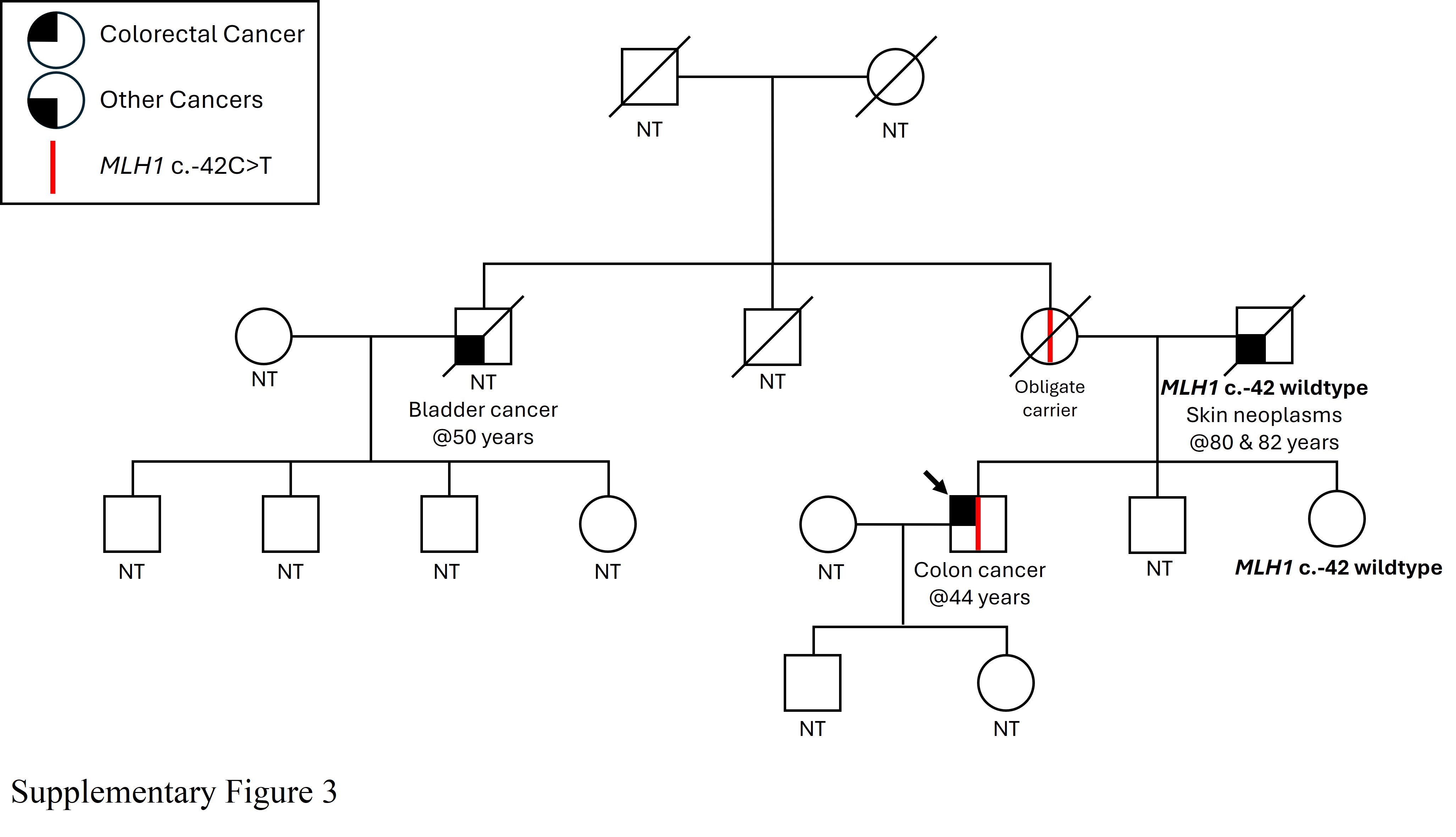

Supplement: Supplementary file 3 — Supplementary Material 3 [file 10689_2025_519_MOESM3_ESM.jpg]

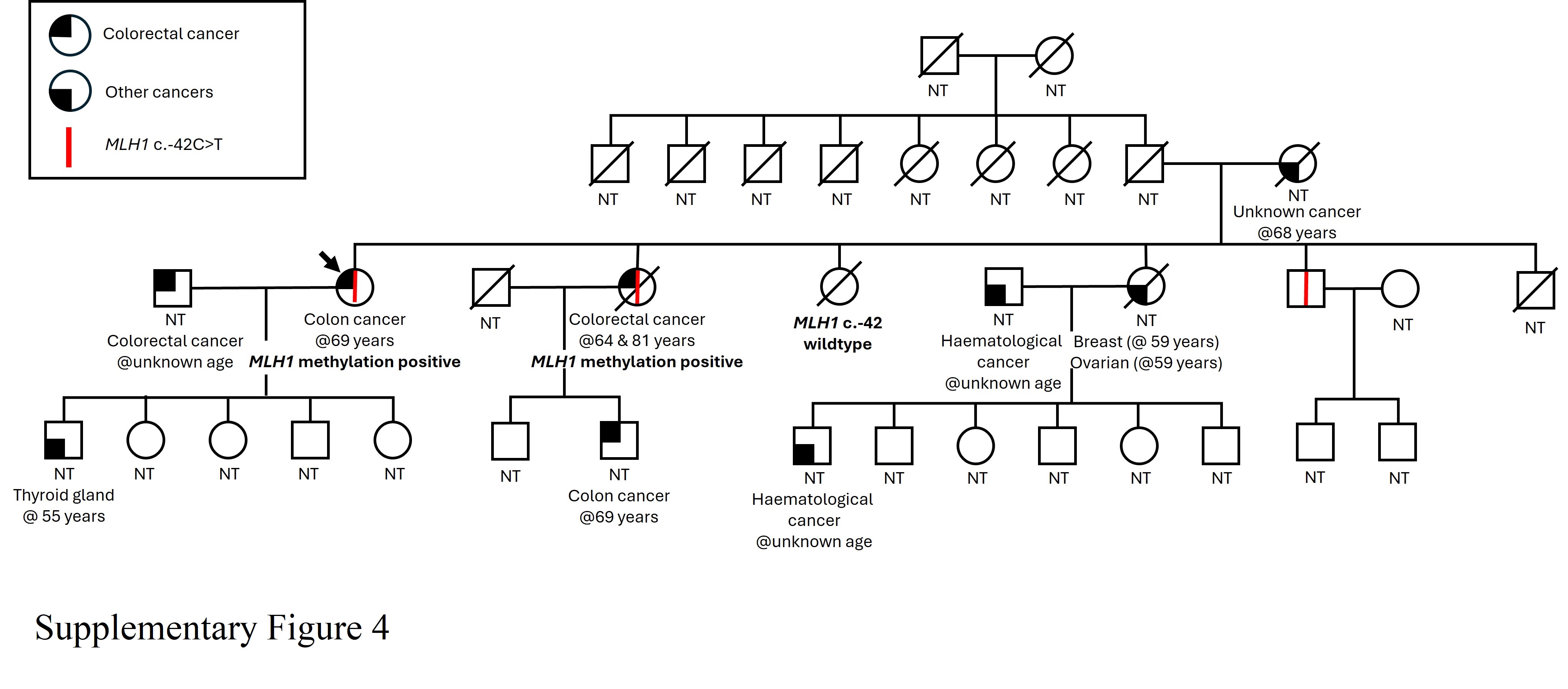

Supplement: Supplementary file 4 — Supplementary Material 4 [file 10689_2025_519_MOESM4_ESM.jpg]

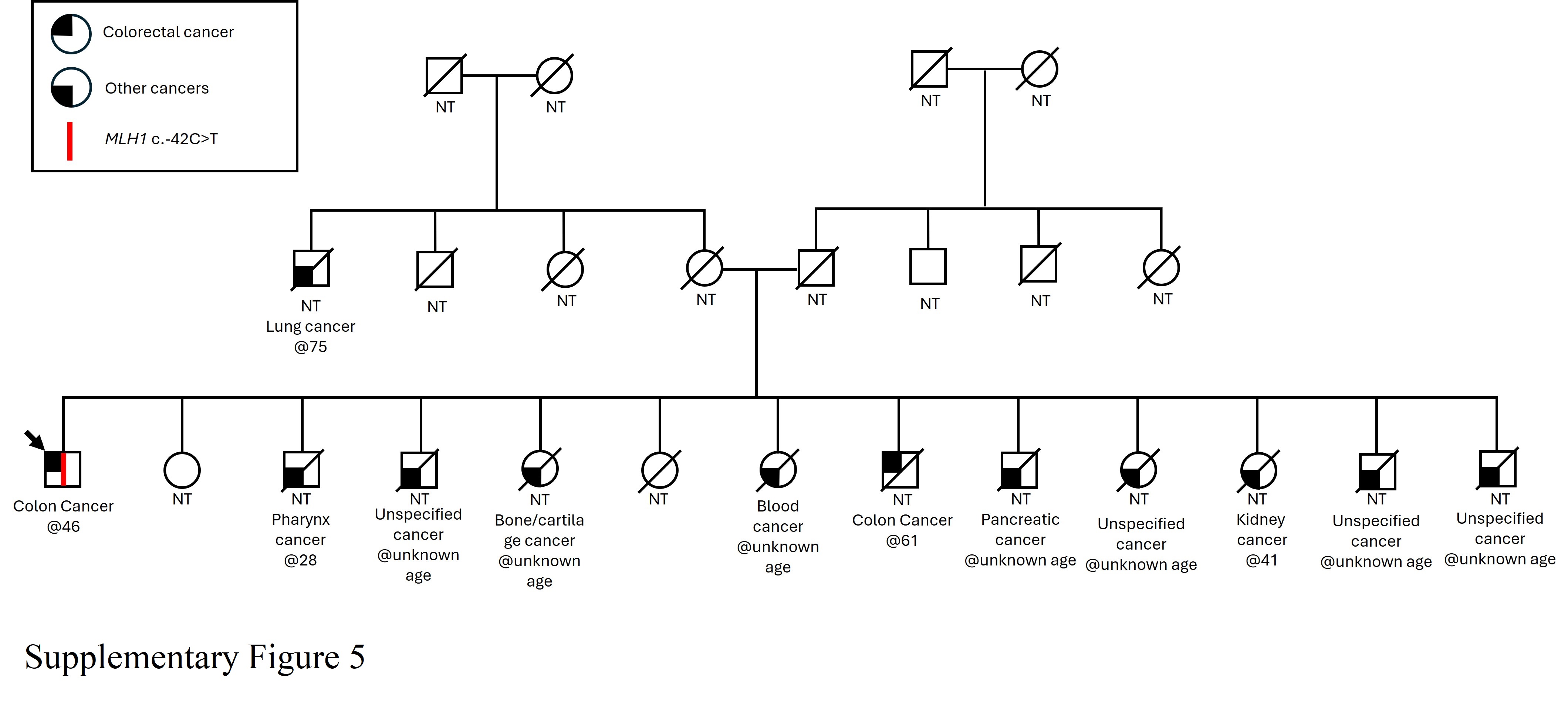

Supplement: Supplementary file 5 — Supplementary Material 5 [file 10689_2025_519_MOESM5_ESM.jpg]
