# Supplementary material for: Germline MLH1 c.-42 C > T is a likely pathogenic variant predisposing to a reduced-penetrance/modified Lynch syndrome phenotype featuring MLH1-methylated cancers
Source: Fam Cancer. 2026 Jan 31;25(1):17. doi: 10.1007/s10689-025-00519-y (PMC12860845; doi:10.1007/s10689-025-00519-y)
Supplement: Supplementary file 6 — Supplementary Material 6 [file 10689_2025_519_MOESM6_ESM.pdf]

Supplementary Table 1. Information on primers used for detecting *MLH1* promoter methylation

Target	Detection platform/technique	Primer	Sequence
MLH1 promoter	methylation-sensitive ddPCR	Forward primer	5’-TTTTCGAGTTTTTAAAAACGAATTAATAGGAA-3’
MLH1 promoter	methylation-sensitive ddPCR	Reverse primer	5’-CTAAATCTCTTCGTCCCTCCCTA-3’
MLH1 promoter	methylation-sensitive ddPCR	Methylated probe	5’-FAM-CGGATAGCGA[EBQ]TTTTTAACGCGTAAGCGT-EBQ-3’
MLH1 promoter	methylation-sensitive ddPCR	Unmethylated probe	5’-HEX-AGTGGATAGT[EBQ]GATTT[LNA T]TAATGT[LNA G]TAAG-EBQ-3’
ACTB	methylation-sensitive ddPCR	Forward primer	5’-AGTAAGTTTTTTGGATTGTGAATTTGTG-3’
ACTB	methylation-sensitive ddPCR	Reverse primer	5’-CCTTAAAAATTACAAAAACCACAACCTAAT-3’

Supplementary Table 2. Haplotyping and ancestry testing based on six microsatellite regions at the *MLH1* locus. The coordinates are in hg38.

			**AUS III-2**		**USA proband**	
**Name**	**Location (*MLH1*)**	**Marker Size**	**allele 1**	**allele 2**	**allele 1**	**allele 2**
D3S11561	36417631	236bp	214	230	216	232
Clen56	36914352	183bp	182	182	182	180
MLH1 c.-93G>A	36993355	SNP rs1800734	A	A	**A**	**G**
MLH1 c.-42C>T	36993506	SNV	T	C	T	C
D3S1611	37002014	233bp	222	232	222	228
MLH1 c.655A>G	37011977	SNP rs1799977	A	A	A	A
Clen57	37096715	222bp	220	218	220	210
Clen58	37281405	191bp	189	185	189	187
D3S3623	37377044	306bp	306	306	306	312

SNP = single nucleotide polymorphism
SNV = single nucleotide variant

Supplementary Table 3 The predicted effect of the *MLH1:* c.-42C>T varaint in transcription factor bindings as estimated using motifbreakR

seqnames	start	end	strand	SNP_id	REF	ALT	varType	motifPos	geneSymbol	dataSource	seqMatch	pctRef	pctAlt	scoreRef	scoreAlt	Refpvalue	Altpvalue	effect	altPos	alleleDiff	alleleEffectSize
chr3	36993505	36993506	-	rs41285097	C	T	SNV	-7;3	ETS2	HOCOMOCO	aaggcacttcCgttgagcatc	0.96640415	0.78172691	4.47658681	3.66696731	6.73E-06	0.00389473	strong	1	-0.8096195	-0.175095644
chr3	36993505	36993506	-	rs41285097	C	T	SNV	-6;3	ETV5	ENCODE-motif	aggcacttcCgttgagcat	0.96079576	0.76145219	4.5733905	3.67303171	1.73E-05	0.00324636	strong	1	-0.9003588	-0.189530823
chr3	36993505	36993506	-	rs41285097	C	T	SNV	-6;3	ETV6	ENCODE-motif	aggcacttcCgttgagcat	0.95689032	0.80383238	5.82808507	4.92740116	1.32E-05	0.00071391	strong	1	-0.9006839	-0.148095734
chr3	36993505	36993506	+	rs41285097	C	T	SNV	-6;3	FLI1	HOMER	aggcacttcCgttgagcat	0.97015792	0.7144589	3.20200091	2.42357007	5.28E-05	0.01615863	strong	1	-0.7784308	-0.236400342
chr3	36993505	36993506	-	rs41285097	C	T	SNV	-4;3	GABPA	ENCODE-motif	gcacttcCgttgagc	1	0.82992126	5.43468834	4.52765886	0	0.00072	strong	1	-0.9070295	-0.166896319
chr3	36993505	36993506	-	rs41285097	C	T	SNV	-7;2	GABPB1+GABPB2	HOCOMOCO	aggcacttcCgttgagcat	0.95605843	0.79543946	5.36617167	4.49364031	1.38E-05	0.0012696	strong	1	-0.8725314	-0.155673632
